# Neutrophil and macrophage influx into the central nervous system are inflammatory components of lethal Rift Valley fever encephalitis in rats

**DOI:** 10.1371/journal.ppat.1007833

**Published:** 2019-06-20

**Authors:** Joseph R. Albe, Devin A. Boyles, Aaron W. Walters, Michael R. Kujawa, Cynthia M. McMillen, Douglas S. Reed, Amy L. Hartman

**Affiliations:** 1 Center for Vaccine Research, University of Pittsburgh, Pittsburgh, Pennsylvania, United States of America; 2 Department of Immunology, University of Pittsburgh School of Medicine, Pittsburgh, Pennsylvania, United States of America; 3 Department of Infectious Diseases and Microbiology, University of Pittsburgh School of Public Health, Pittsburgh, Pennsylvania, United States of America; Division of Clinical Research, UNITED STATES

## Abstract

Rift Valley fever virus (RVFV) causes severe disease in livestock concurrent with zoonotic transmission to humans. A subset of people infected with RVFV develop encephalitis, and significant gaps remain in our knowledge of how RVFV causes pathology in the brain. We previously found that, in Lewis rats, subcutaneous inoculation with RVFV resulted in subclinical disease while inhalation of RVFV in a small particle aerosol caused fatal encephalitis. Here, we compared the disease course of RVFV in Lewis rats after each different route of inoculation in order to understand more about pathogenic mechanisms of fatal RVFV encephalitis. In aerosol-infected rats with lethal encephalitis, neutrophils and macrophages were the major cell types infiltrating the CNS, and this was concomitant with microglia activation and extensive cytokine inflammation. Despite this, prevention of neutrophil infiltration into the brain did not ameliorate disease. Unexpectedly, in subcutaneously-inoculated rats with subclinical disease, detectable viral RNA was found in the brain along with T-cell infiltration. This study sheds new light on the pathogenic mechanisms of RVFV encephalitis.

## Author summary

Rift Valley fever is a disease of livestock and humans that occurs periodically in Africa and parts of the Middle East. People infected with Rift Valley fever virus can develop different clinical outcomes, including hemorrhagic fever or encephalitis. Understanding of the pathophysiological mechanisms of encephalitis has been a challenge due to inadequate animal models of neurological disease. We used a rat model of Rift Valley fever encephalitis to understand the mechanisms underlying lethal neurological disease compared to subclinical infection. The primary immune cell types that infiltrate the brains of rats with lethal encephalitis were neutrophils and macrophages. Prevention of neutrophil migration into the brains did not prevent disease. These results provide new insight into the contrast between lethal and subclinical disease in an immunocompetent rodent model of Rift Valley fever encephalitis.

## Introduction

Africa suffers from periodic outbreaks of Rift Valley fever (RVF), a disease of both livestock animals and humans. Abnormally heavy rainfall in 2018 led to cases of RVF in animals and people in the countries of South Sudan, Uganda, Gambia, Rwanda, Kenya, and the archipelago of Mayotte [1–3]. Concern that the spread of RVFV in competent mosquito vectors could lead to emergence beyond its current endemnicity in Africa and the Arabian Peninsula has prompted the World Health Organization to include RVFV as a pathogen of concern and a priority for research and development [4].

Not all human infections with RVFV are obtained through mosquito bite; another mechanism of infection of humans with RVFV is through handling infected livestock or consuming milk or meat from sick animals [5, 6]. Most humans infected with RVFV survive the infection but experience generalized symptoms of fever, headache, nausea, vomiting, and body pains [7]. A small number of patients develop rapidly-progressing hemorrhagic fever with significant liver necrosis, while other patients may develop meningoencephalitis [8]. Neurological signs consist of hypersalivation, confusion, coma, hallucination, and signs of meningeal irritation [9]. Both the hemorrhagic/hepatotropic and encephalitic manifestations of RVF have high mortality rates in humans (~50% for hospitalized patients) [8]. Development of severe disease outcomes is associated with exposure to RVFV when handling infected animals [6, 10]. In both laboratory animals and humans, vaccines and therapeutic drugs that can protect from hepatotropic and hemorrhagic RVF often fail to protect from neurological manifestations [11–15]. Therefore, a more detailed understanding of the neuropathogenic mechanisms of RVF is merited.

Infection of adult Lewis rats with a fully virulent strain of RVFV by inhalation provides a reproducible model of lethal viral encephalitis [16]. After aerosol infection with RVFV strain ZH501, Lewis rats develop neurological signs and are moribund within 7–8 days. Our recent study demonstrated that widespread permeability of the brain vasculature in lethally-infected rats occurred at the end of the disease process, from 5 days post-infection (dpi) onwards [17]. We found that RVFV was replicating within the brain prior to changes in brain vascular permeability. Previous studies have not determined what types of immune cells infiltrate the brain during the course of RVF encephalitis in rats. To address this, we will characterize the immune populations present during RVFV encephalitis using flow cytometry and fluorescent imaging. Unlike aerosol (AERO) exposure, infection of Lewis rats by subcutaneous (SC) injection of the virus results in a sub-clinical infection [16, 18]. This is in contrast to mice, where there was no difference in survival between SC and AERO exposure routes, although AERO exposed mice developed more severe neuropathology [15]. Here we report our efforts to utilize the difference in disease outcome of Lewis rats infected by SC or AERO to shed light on the pathogenic mechanisms resulting in lethal encephalitic disease. Our main findings are that neutrophils and macrophages are the primary cell types infiltrating the brain during lethal RVFV encephalitis. When neutrophils were prevented from entering the CNS, the disease outcome after AERO infection was not altered. In comparison, subclinical disease after SC infection is associated with detectable viral RNA in the brain during the course of infection, despite no demonstrable clinical signs. This study provides important knowledge about the pathogenic events leading to lethal RVF encephalitis.

## Results

### Infection route determines survival of Lewis rats

**Fig 1:**
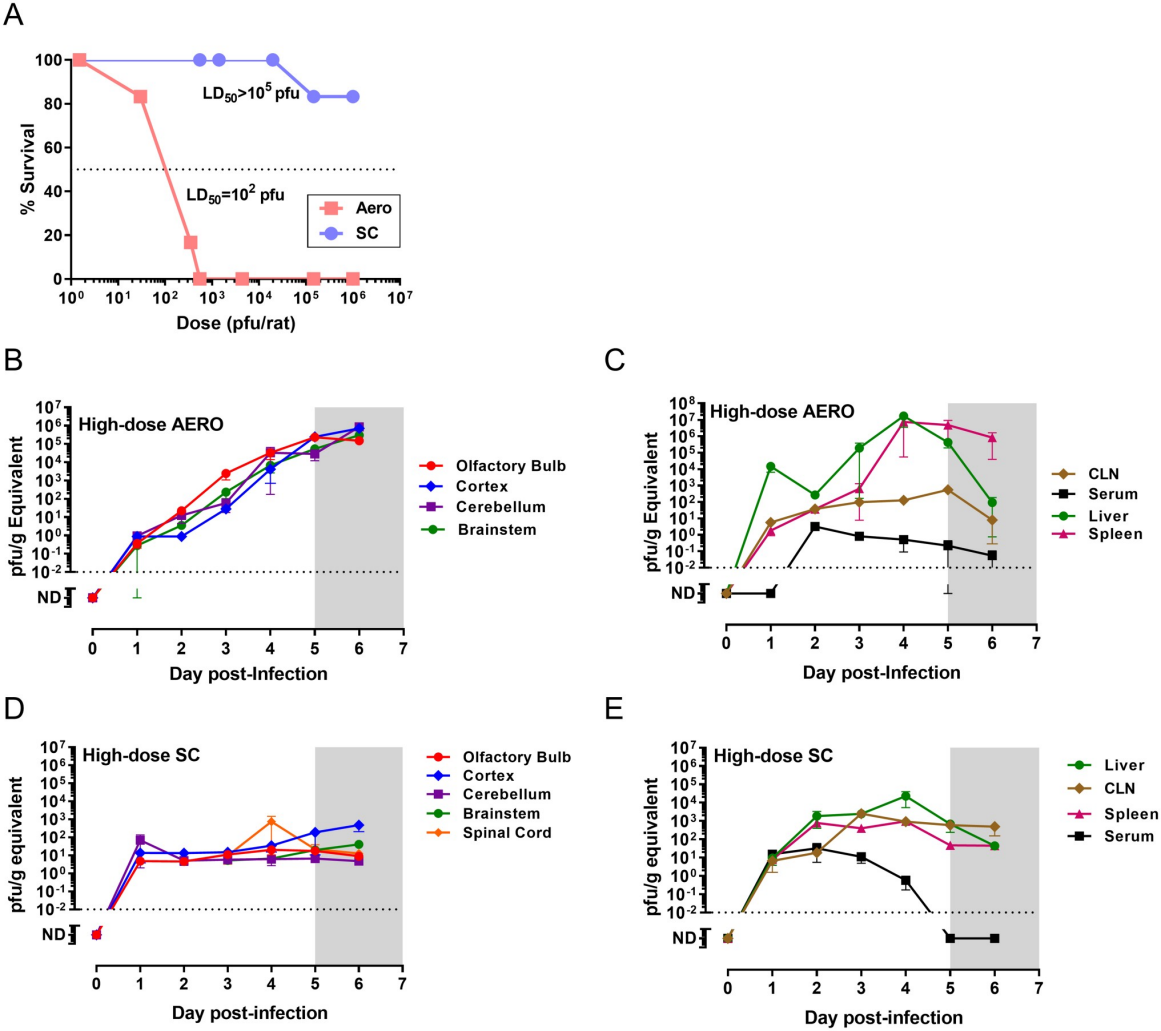
Rat survival and spread of RVFV throughout tissues after SC or AERO infection. Rats were challenged with RVFV ZH501 via AERO or SC. (A) Comparison of survival at the indicated exposure doses (n = 6–12 rats per dose group). (B-E) vRNA (measured by q-RT-PCR and expressed as pfu/g equivalents) was measured in each indicated tissue (n = 3–7 rats/group). (B) CNS tissues from AERO-infected rats (C) peripheral tissues from AERO-infected rats. (D) CNS tissues from SC-infected rats (E) peripheral tissues from SC-infected rats. Dotted line represents limit of detection of the q-RT-PCR assay. Shaded grey box (5–7 dpi) represents clinical window during which AERO-infected Lewis rats display signs of illness.

Lewis rats become infected but do not develop clinical signs of disease after SC infection with the pathogenic wild-type ZH501 strain of RVFV [16, 18]. This is in stark contrast to what happens after AERO infection in the same strain of rat (Fig 1A). Lewis rats succumb to encephalitic disease within 6–8 days after AERO infection, depending upon the dose (LD_50_ = 112 pfu) [16]. Signs of illness (fever, weight loss and neurological signs) begin at 5 days post-infection (dpi) and continue until euthanasia criteria are met [16, 19]. In this study, we compare the course of sub-lethal (SC) and lethal (AERO) infection in Lewis rats to better understand the mechanisms separating these opposing clinical outcomes.

### Spread of virus throughout tissues

Our previous study showed that after AERO infection of Lewis rats with 1x10^3^ pfu of RVFV, virus progresses through the brain from the olfactory bulb posteriorly to the brain stem and spinal cord [17]. Here, we infected Lewis rats with a higher dose of RVFV (3x10^4^ pfu) and compared it to SC infection (1x10^5^ pfu) to understand viral spread through the animals after inoculation at different locations (Fig 1B–1E). Each day after infection from 1–6 dpi, rats were euthanized to collect tissues (n = 3–7 per time point). Viral RNA (vRNA) was measured by qRT-PCR in CNS and peripheral tissues. Given the higher AERO virus dose compared to the previous study, the temporal spread of virus through the CNS was compressed, although there were higher titers in the anterior CNS tissues such as the olfactory bulb (Fig 1B).

We hypothesized that the reason Lewis rats do not develop illness after SC inoculation is because virus replication is controlled within the periphery and does not reach the brain. Unexpectedly, we found detectable vRNA in all CNS regions of SC-inoculated rats throughout the time-course observed (1–6 dpi; Fig 1D). vRNA titers in the brains of SC-inoculated rats did not rise during the course of infection, but remained at consistent levels as compared to AERO infection, which resulted in a steady increase in vRNA in all brain regions over the duration of the experiment up to 1x10^6^ pfu/ml equivalents. The detection of vRNA in the brains of SC-infected rats was consistent over 2 separate serial-sacrifice experiments using 3 rats/time point for each experiment (n = 6 rats/time point). Because we were able to detect vRNA in all of the brain samples across all of the time points at levels 3-logs above the q-PCR cutoff suggests these are not a result of cross-contamination of the PCR reaction.

Attempts to isolate infectious virus from brain cortex samples from both AERO- and SC-infected rats by passage twice on Vero cells resulted in minimal cytopathic effect (CPE). In passaged samples obtained from early infection (1–3 dpi), vRNA was variably detected in the passaged cultures from both AERO- and SC-infected rats (2 of 9 and 1 of 9 passaged cultures were vRNA+, respectively). Passaged cortex samples from SC-infected rats at later time points (4–7 dpi) yielded more consistent detection of vRNA in the passaged cultures (7 of 12 cultures were vRNA+; 58%). Our previous study compared infectious virus and vRNA levels within the brains of AERO-infected rats and found infectious virus detectable by plaque assay at 5 dpi and onwards, while vRNA was detectable by 1 dpi [19]. Here, we attempted to culture infectious virus rather than plaque it, assuming that this method would be more sensitive to isolating very low levels of infectious virus. We were surprised at the difficulty culturing infectious virus from the early brain samples, particularly in the early samples from the AERO group because we can reproducibly detect substantial levels of vRNA and we can also visualize the virus by IF and IHC. Difficulty culturing may be due to the homogenization procedure, performed using an Omni tissue homogenizer, which may render low levels of infectious virus difficult to culture, whereas effects of homogenization on higher starting levels of infectious virus are not as obvious. Alternatively, culture of low levels of virus may be hampered by factors in the brain homogenate or the virus may remain cell-associated. Given that 58% of the 4–7 dpi samples from SC-infected rats were vRNA+ after passage, there appears to be virus within the brain of these animals. It is not known if this is replicating infectious virus that could reactivate at a later date and cause disease in the rats.

Distribution of vRNA in cervical lymph node (CLN) was similar after either route of infection; both routes has detectable virus in the CLN at 1 dpi (Fig 1C and 1E). vRNA was detectable in serum of SC-infected rats at 1 dpi but not until 2 dpi in the AERO-infected rats. Approximately 100-fold more vRNA was found in the liver and spleen of the AERO-infected rats compared to SC-infected.

### Granulocytosis is a feature of end-stage RVF disease after AERO-infection

**Fig 2:**
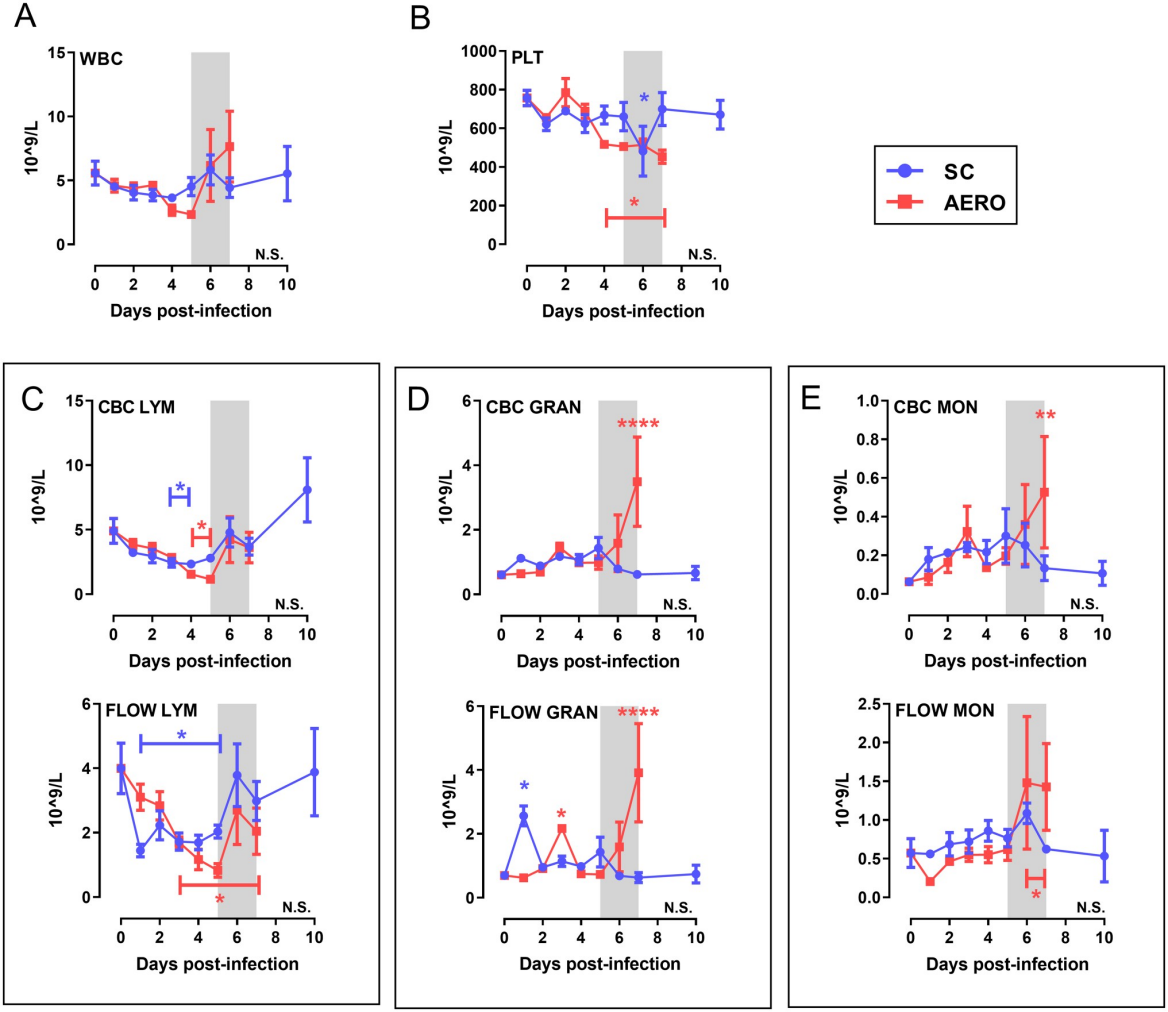
Granulocytosis is a feature of end-stage RVF disease after AERO-infection. 18-parameter complete blood count (CBC) and multicolor flow cytometry were performed on whole blood at each time point. (A) Total white blood cells (WBC) and (B) platelets (PLT). (C-E) Paired graphs show a comparison between data obtained from CBC (top) and flow cytometry (bottom). (C-E) Total # of each indicated cell type. Data from rats infected SC with 1x105 pfu/rat and euthanized at 10 dpi is included for comparison. Shaded grey box represents clinical window of AERO-infected rats. N = 3–6 rats/timepoint. LYM = lymphocytes; GRAN = granulocytes; MON = monocytes. Gating strategy shown in S3 Fig. Asterisks above symbols indicate significance of individual time points compared to uninfected (N.S., not significant; *, P < 0.05; **, P < 0.01; ***, P < 0.001; ****, P < 0.0001). Asterisk above a bar indicates significance over the encompassed data points.

To compare differences in disease course between SC and AERO exposure, whole blood was analyzed by complete blood count (CBC) analysis and flow cytometry (S3 Fig). A comparison of parameters obtained from both techniques showed congruence and validation for both analysis methods. Total white blood cell (WBC) numbers did not significantly differ from pre-infection (baseline) after either infection route (Fig 2A), but there were changes in specific cell populations over time. Thrombocytopenia was sustained after AERO but not SC infection (Fig 2B). Unlike AERO-infection, granulocytosis was limited after SC infection (Fig 2C and 2D). Lymphopenia (measured by both methods) occurred by 1 dpi through 5 dpi in both groups, but the SC-infected rats returned to baseline levels quicker while AERO-infected rats only partially recovered. Lymphopenia and granulocytosis are documented findings during lethal RVF infection, in both animal models and human clinical samples [8, 19–22]. These data provide a direct comparison of changes in the peripheral blood between SC vs AERO infection routes and also serve to validate both the CBC analysis and flow cytometry as complementary methods for blood cell typing in rats.

### Neutrophils and macrophages are the predominant cell types infiltrating the brains of AERO-infected rats

**Fig 3:**
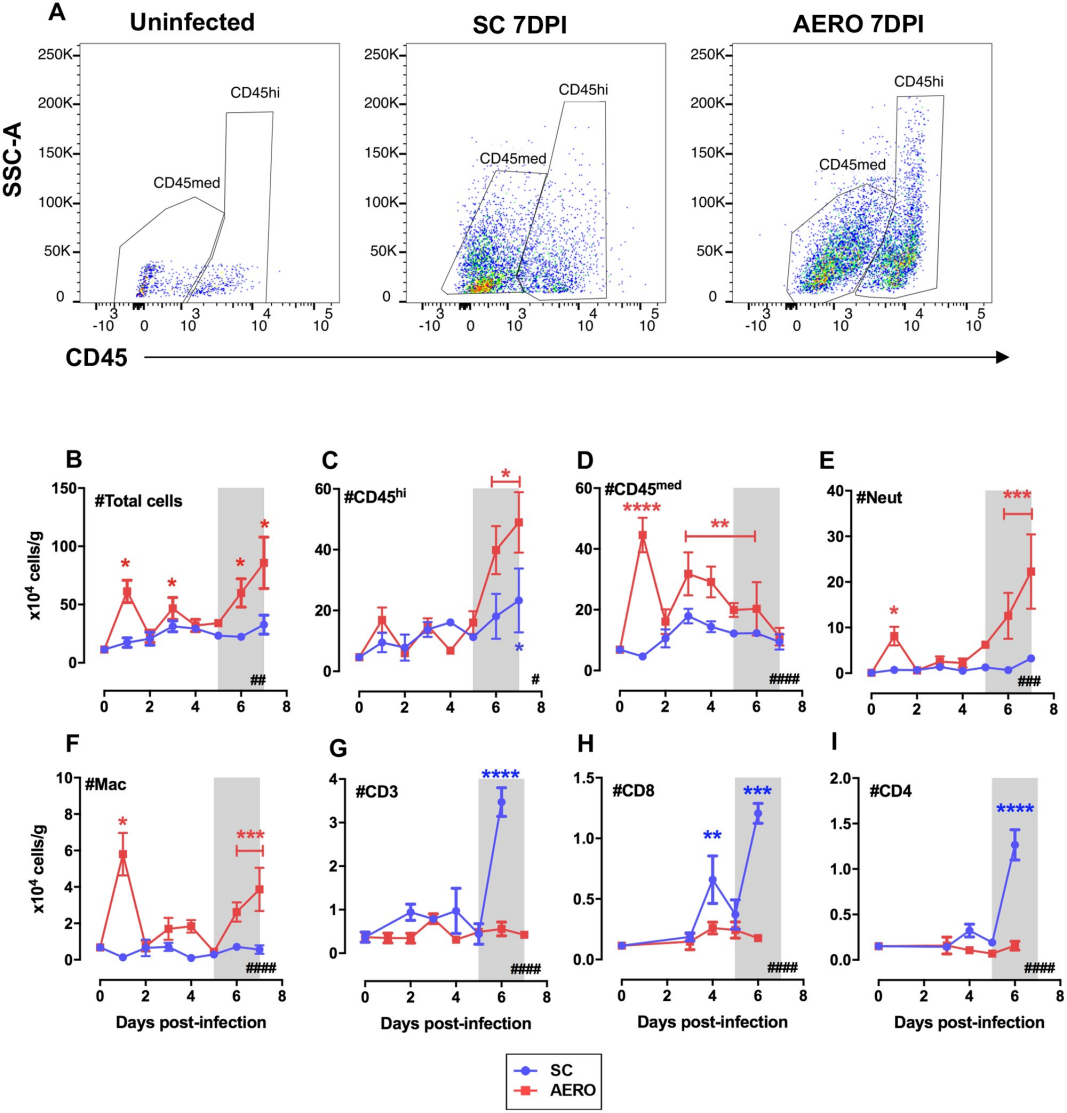
Leukocyte infiltration into the brains of RVFV-infected rats. Characterization of cell infiltrates into rat brains was done by flow cytometry. (A) Representative examples of CD45 expression on live cells obtained from uninfected (left), SC-infected (middle), and AERO-infected (right) rats as characterized by flow cytometry. (B) Total cell counts obtained by counting live cells on a hemocytometer after ficoll-gradient isolation. (C) CD45^hi^ cells, which represent either infiltrating leukocytes or activated microglia, (D) CD45^med^ cells represent resting microglia (E) Neutrophils (Neut) as identified by RP-1+, CD11b+, CD45+, (F) Macrophages (Mac) as identified by CD163+, CD11b+, CD45+, (G) CD3+, (H) CD8+, and (I) CD4+ cells. N = 3–7 rats/timepoint. Day 0 time point represents mock-infected rats. The # in the bottom right corner indicates significance by 2-way ANOVA (#, P < 0.05; ##, P < 0.01; ###, P < 0.001; ####, P < 0.0001); asterisks above symbols indicate significance of individual time points compared to uninfected (*, P < 0.05; **, P < 0.01; ***, P < 0.001; ****, P < 0.0001). Asterisk above a bar indicates significance over the encompassed data points.

Leukocytes were isolated from rat brain hemispheres harvested each day after infection to determine the phenotype and timing of infiltrating cells by flow cytometry. Expression levels of CD45 distinguishes resting, resident microglia (CD45^med^) from activated microglia or peripheral leukocytes (CD45^hi^) [23]. In a normal, uninfected rat brain, two CD45+ populations are distinguishable, with the majority being CD45^med^ phenotype (Fig 3A, left panel). In contrast, the brain from an AERO-infected rat at end-stage disease contains many CD45^hi^ cells, and they are more granular and complex (larger SSC-A) (Fig 3A, right panel). SC-infected rat brains harvested at 7 dpi were of an intermediate phenotype; both CD45 populations were detectable (Fig 3A, middle panel).

Using total CD45+ cells, we characterized peripheral macrophages infiltrating the CNS as CD163+, neutrophils as RP-1+ CD11b+, and microglia as Iba-1+ (S4 Fig). After live-dead exclusion, vital brain cell gating, and singlet inclusion, all parameters are expressed as the number of cells per gram of brain tissue based on back calculating cell counts using a hemocytometer with the percentage of total CD45+ cells.

Upon examination of brain samples harvested daily over the entire time course, from 1–7 dpi, the total live cell counts increased during the course of infection in AERO-infected rats (Fig 3B). Unexpectedly, the AERO-infected rats had an early transient increase in CD45+ cell numbers on 1 dpi comprised of both neutrophils and macrophages, possibly suggesting proliferation of resting microglia and early leukocyte entry to the CNS may be detrimental to the clinical outcome (Fig 3D–3F). After 1 dpi, the number of cells in AERO-infected brains remained near pre-infection levels until a major increase in cells at 5 dpi and after. This corresponds to increased vascular permeability observed in AERO-infected Lewis rats from 5 dpi onwards in our previous study [17]. The cells in end-stage AERO-infected brains were primarily CD45^hi^ (10-fold overall increase from baseline) and consisted predominantly of neutrophils (250-fold change) and macrophages (5-fold change), with very little change in T-cell numbers. The CD45^hi^ cells may be macrophages infiltrating from the periphery or resident brain microglia that became activated and increased CD45 expression.

Due to the subclinical disease, we did not expect to see leukocyte infiltration into the brains of SC-infected rats. This not the case, as we observed influx of cells into the CNS of SC-infected rats, albeit not as great as was seen with AERO-infected rats. There was a slight increase in CD45^hi^ cells into the SC-infected brains that was significant at 7 dpi (Fig 3C). There was not an early change in either CD45^med^ or CD45^hi^ cells at 1 dpi as was seen in the AERO infected rats, nor was there a dramatic increase in neutrophils nor macrophages at 6–7 dpi. There was, however, a sharp increase in T-cells (both CD4 and CD8) at 6 dpi (Fig 3G–3I).

Taken together, immune infiltration into the brain was observed after both SC and AERO infection, but the AERO-infected rats had a more dramatic infiltration of neutrophils and macrophages at end-stage disease after the vasculature became permeable [17]. Conversely, a late T-cell infiltration as associated with rats that survive SC infection.

### Early Th2 and TH17 cytokine responses are associated with survival from SC-infection

**Fig 4:**
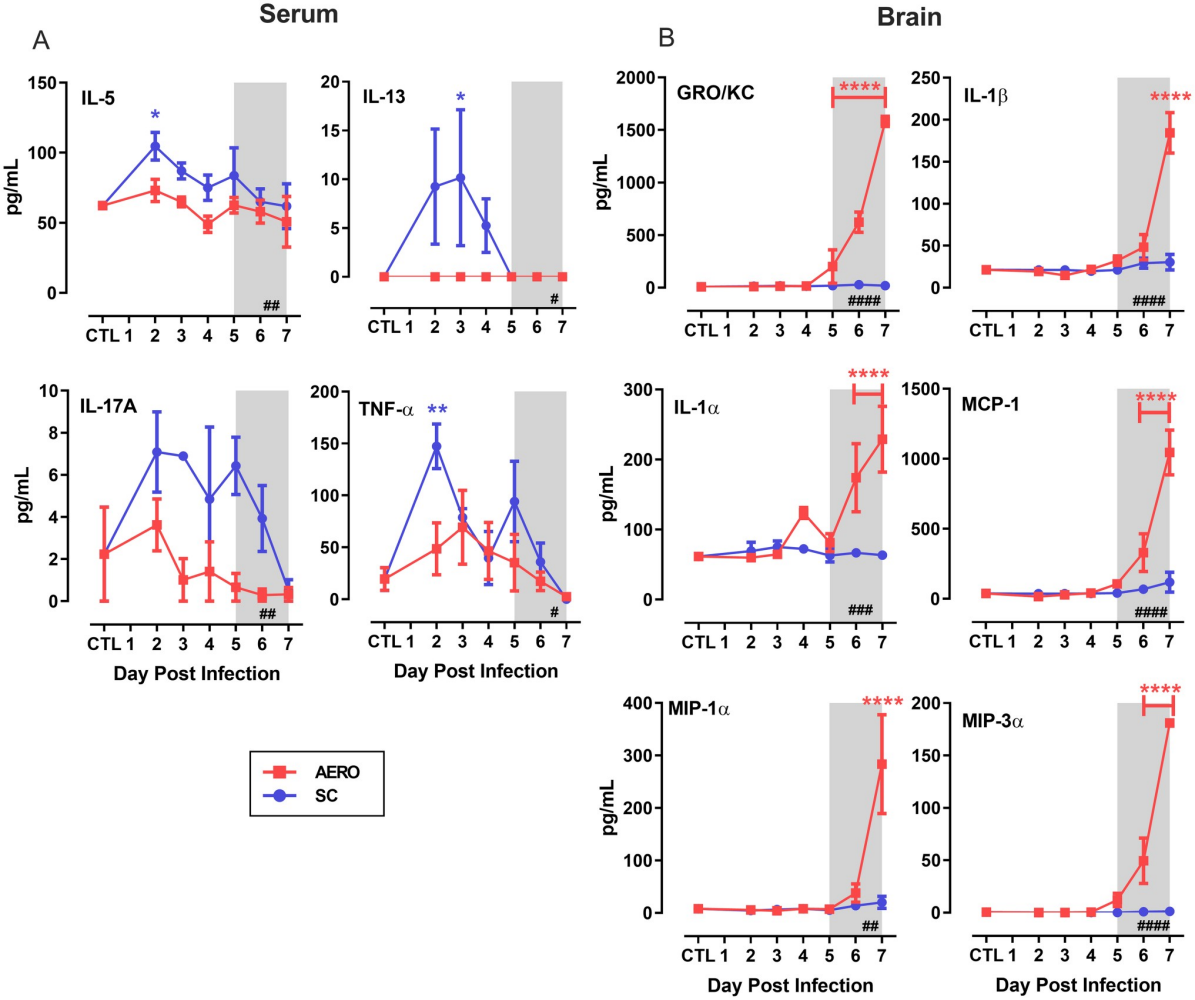
Early serum cytokine responses occur in SC-infected rats compared to a late cytokine/chemokine storm in the brain of AERO-infected rats. Cytokines were assessed by a 22-plex Luminex kit. (A) Select serum cytokines. (B) Select cytokines in homogenized brain tissue. CTL represents mock-infected rats. For statistical analysis, 2-way ANOVA with multiple comparisons was performed (see Methods section). The # in the bottom right corner indicates significance by 2-way ANOVA (N.S., not significant; #, P < 0.05; ##, P < 0.01; ###, P < 0.001; ####, P < 0.0001); asterisks above symbols indicate significance of individual time points compared to uninfected (*, P < 0.05; **, P < 0.01; ***, P < 0.001; ****, P < 0.0001). Asterisk above a bar indicates significance over the encompassed data points.

Inflammatory cytokine expression occurs within the sera and brains of AERO-infected rats that die of encephalitis [19]. We used a multiplex cytokine panel to compare samples from AERO and SC-infected rats to provide insights as to differential cytokine responses that are associated with non-lethal disease. Within the sera of SC-infected rats, we found elevated levels of the Th2 cytokines IL-5 and IL-13, the Th17 cytokine IL-17A, and TNF-α (Fig 4A). AERO-infected rats had no significant changes in any of these cytokines in the serum. However, the brains of AERO-infected rats had high levels of inflammatory chemokines IL-1α, IL-1β, Gro/KC, MCP-1, MIP-1α, and MIP-1β end stage disease (Fig 4B). These data are similar to what we observed in a monkey model of RVF encephalitis, where an early cytokine response in the serum was associated with survival, whereas late cytokine storm in the brain occurred during lethal (AERO) disease [20].

### Human and rat microglia and neurons are susceptible to RVFV infection

**Fig 5:**
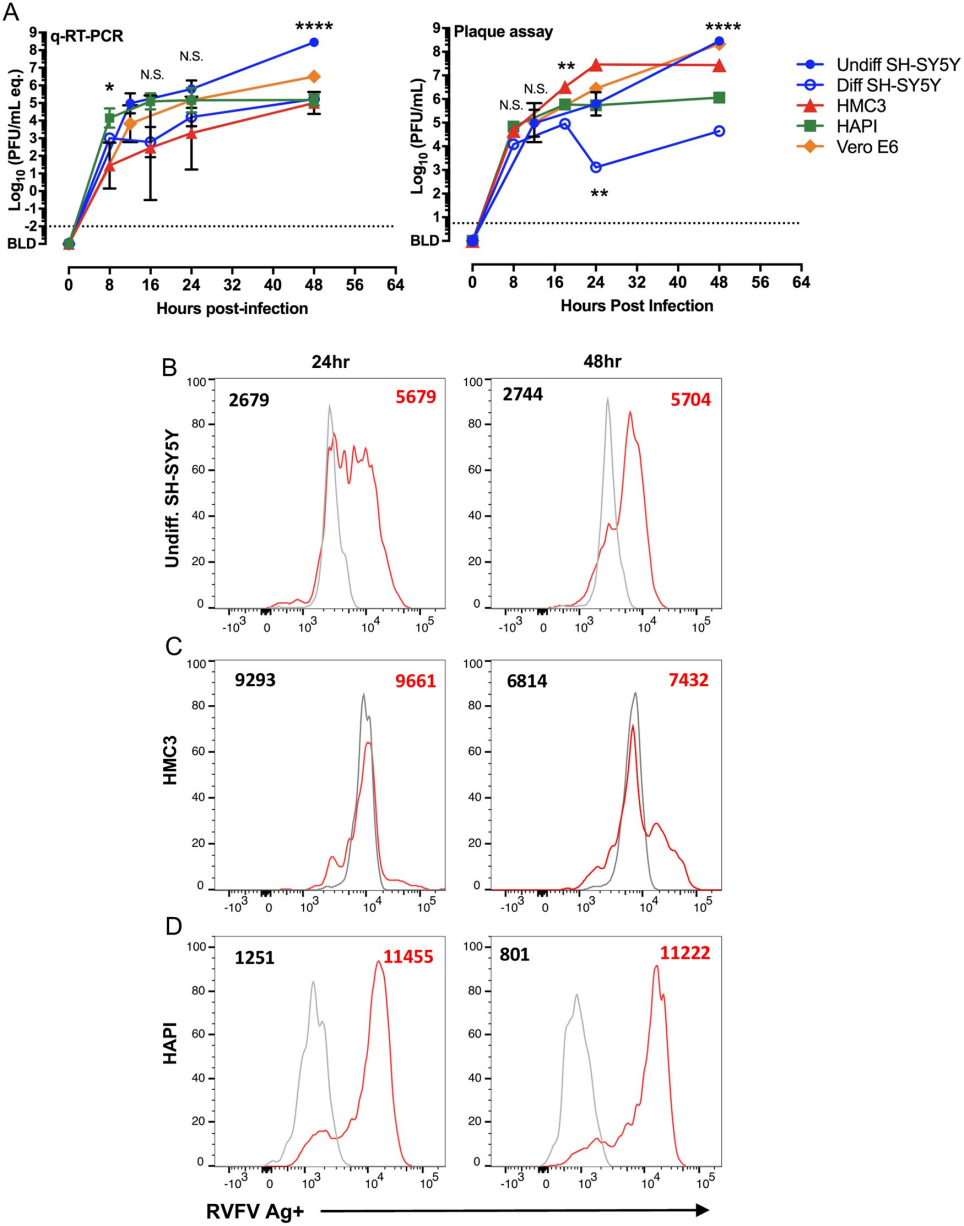
Replication of RVFV in CNS cell lines and detection of infected cells by flow cytometry. (A) vRNA production (left) and infectious virus (right) produced from the indicated cell lines after infection at MOI = 1. One-way ANOVA was used to determine statistical significance between the cell lines at each time point. Significance indicated by asterisks. RVFV antigen was detected in (B) undifferentiated SH-SY5Y cells (C) HMC3, and (D) HAPI cells using intracellular flow cytometry. Uninfected cells (gray) were stained at the same time as the infected cells (red). Numbers indicated MFI of RVFV antigen staining.

To determine the *in vitro* susceptibility of CNS cell types to RVF infection, human neurons (SH-SY5Y), human microglia (HMC3), rat microglia (HAPI), and Vero cells (for comparison) were infected at a multiplicity of infection (MOI) of 1.0. Viral growth over time was determined by qRT-PCR and viral plaque assay over 48 hours. SH-SY5Y were either undifferentiated (immature) or differentiated with retinoic acid. RVFV replicated to high levels in each cell line, with immature SH-SY5Y cells producing the highest titers by 48hpi (Fig 5A). There was a 4-log spread of virus titers by both assays across the different cell lines by 48 hpi. This emphasizes the broad tropism of RVFV and its ability to replicate in different cell types including microglia and neurons. The immature SH-SY5Y cells were significantly more permissive for infection and virus production than the differentiated neurons, indicating that differentiation status may play a role on virus tropism.

We developed an intracellular flow cytometry method to identify infected cells using a monoclonal antibody to the viral Gn glycoprotein. This method was validated using RVFV-infected Vero cells and other permissive CNS cell lines (Fig 5B–5D). The Gn-specific antibody was able to detect viral antigen within each cell type based on median fluorescent intensity (MFI) (Fig 5B–5D).

### Detection of viral antigen in microglia and neutrophils during lethal RVF encephalitis

**Fig 6:**
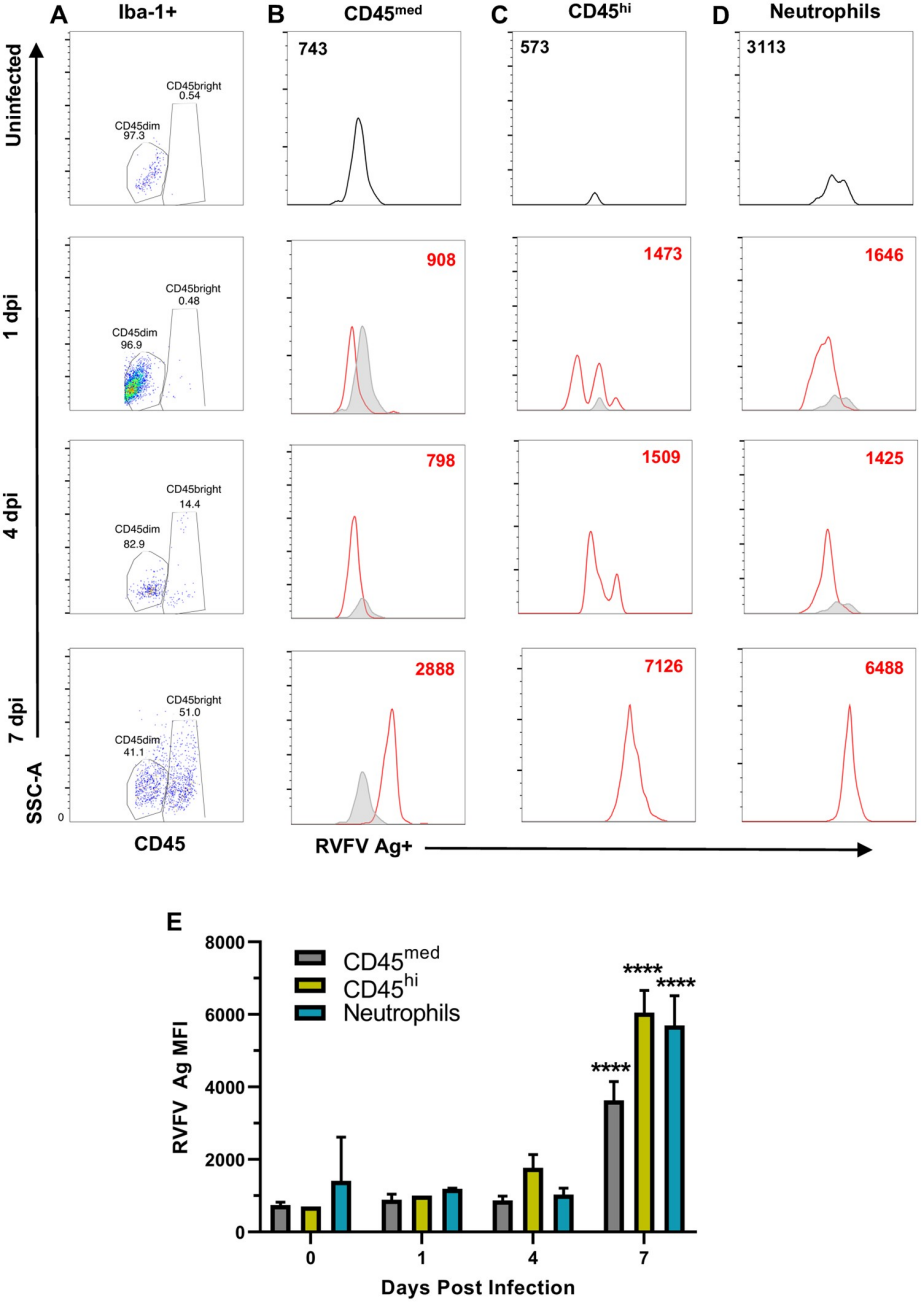
Detection of RVFV-infected cells within rat brains. Representative flow cytometry plots of brain cells isolated from AERO-infected rats. Live cells were first gated on Iba-1 then assessed for CD45 expression. (A) All Iba-1+ cells showing differential expression of CD45. Viral antigen within (B) CD45med, (C) CD45hi, and (D) neutrophils. Cells isolated from uninfected rat brains (gray) are compared to infected rat brains (red) at the indicated time points. The corresponding MFI of viral antigen staining is indicated by the number within the histogram. (E) Comparison of MFI of RVFV Ag staining within the indicated cell populations (n = 2–4 samples/time point). Statistical significance determined by 2-way ANOVA with multiple comparisons. MFI from all 7 dpi samples were statistically significant compared to 0,1,4 dpi.

For the remainder of this study, we focused on AERO-infected rats. Intracellular flow cytometry was used to detect viral antigen within freshly obtained leukocytes from AERO-infected rat brains at 1, 4, and 7 dpi (Fig 6). We isolated brain leukocytes as described above, performed live/dead, singlet inclusion, and vital brain cell gating, followed by gating on Iba-1+ cells (S2 Fig). CD45 expression was used to distinguish resting microglia (CD45^med^) from activated microglia (CD45^hi^) (Fig 6A). Viral antigen was detectable in Iba-1+CD45^med^ cells, Iba-1+CD45^hi^ cells, and neutrophils by 7 dpi (Fig 6B and 6D), at which point nearly all of the Iba-1+ cells contained viral antigen. Additionally, the CD45^hi^ cells were of increased complexity as measured by SSC-A (Fig 6A), suggesting a morphological distinction from those events in the CD45^med^ gate. This change may be the result of microglia changing from a ramified state to an ameboid state, which occurs upon microglial activation [24].

### Visualizing RVFV infection in the brains of AERO-infected rats

**Fig 7:**
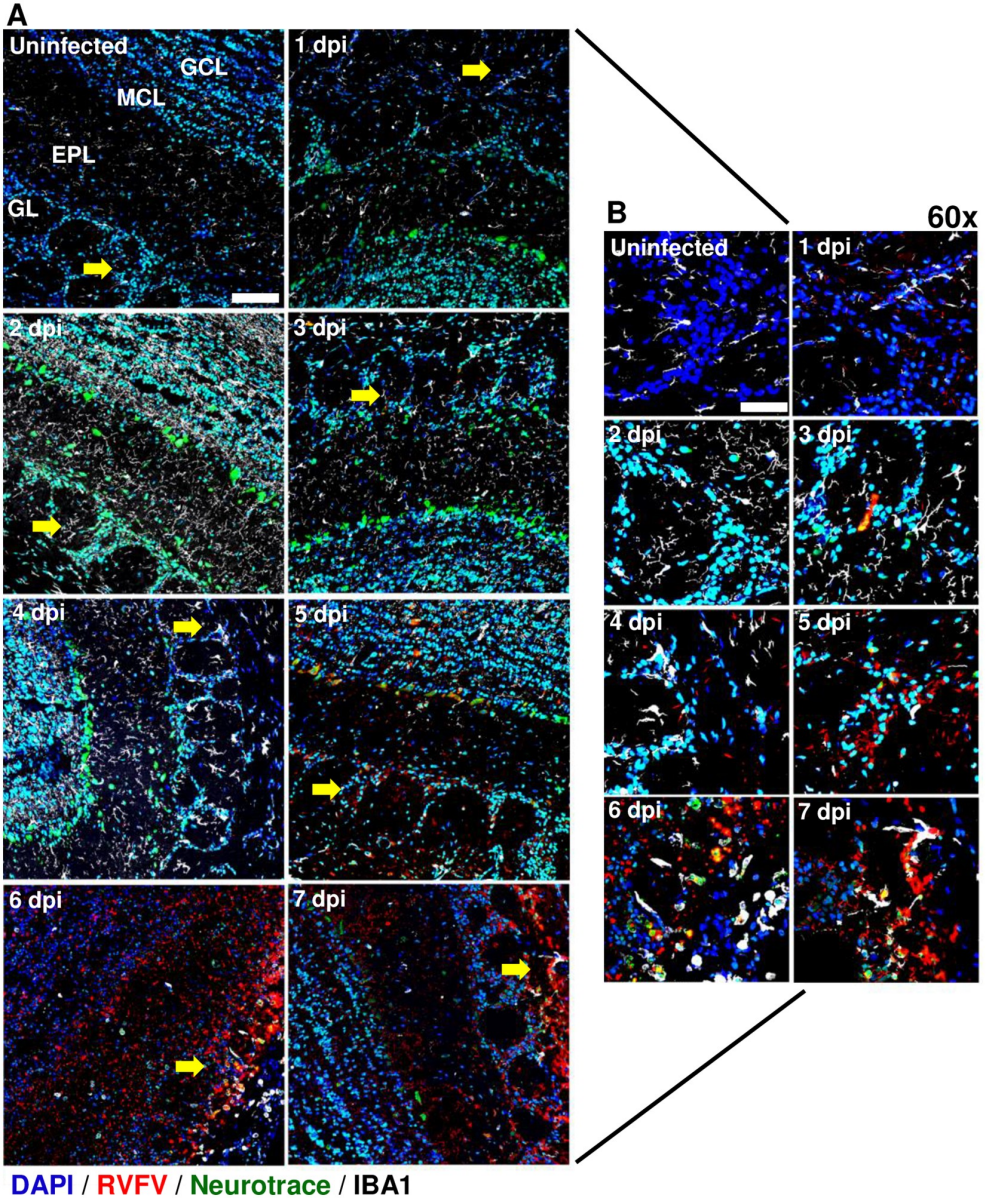
Visualization of RVFV infection in the olfactory bulb. AERO-infected Lewis rat olfactory bulbs show evidence of increased viral RNA within the glomerular layer from 1 dpi to 7 dpi. RVFV RNA was detected by in situ hybridization immunofluorescence (ISH-IF) (red). Samples were co-stained with an antibody for microglia (IBA-1; white), a Nissl body dye (Neurotrace; green), and nuclear counterstain (DAPI; blue). (A) Uninfected 40x micrograph labeled: GL; glomerular layer, EPL; external plexiform layer, MCL; mitral cell layer, GCL; granule cell layer. 2x2 field large images at 40x magnification were stitched using Nikon Elements software (scale bar = 100um). Yellow arrows indicate areas of viral RNA detection within the glomerular layer that is magnified in (B). (B) Max intensity projection images of the glomerular layer taken at 60x (scale bar = 50um).

**Fig 8:**
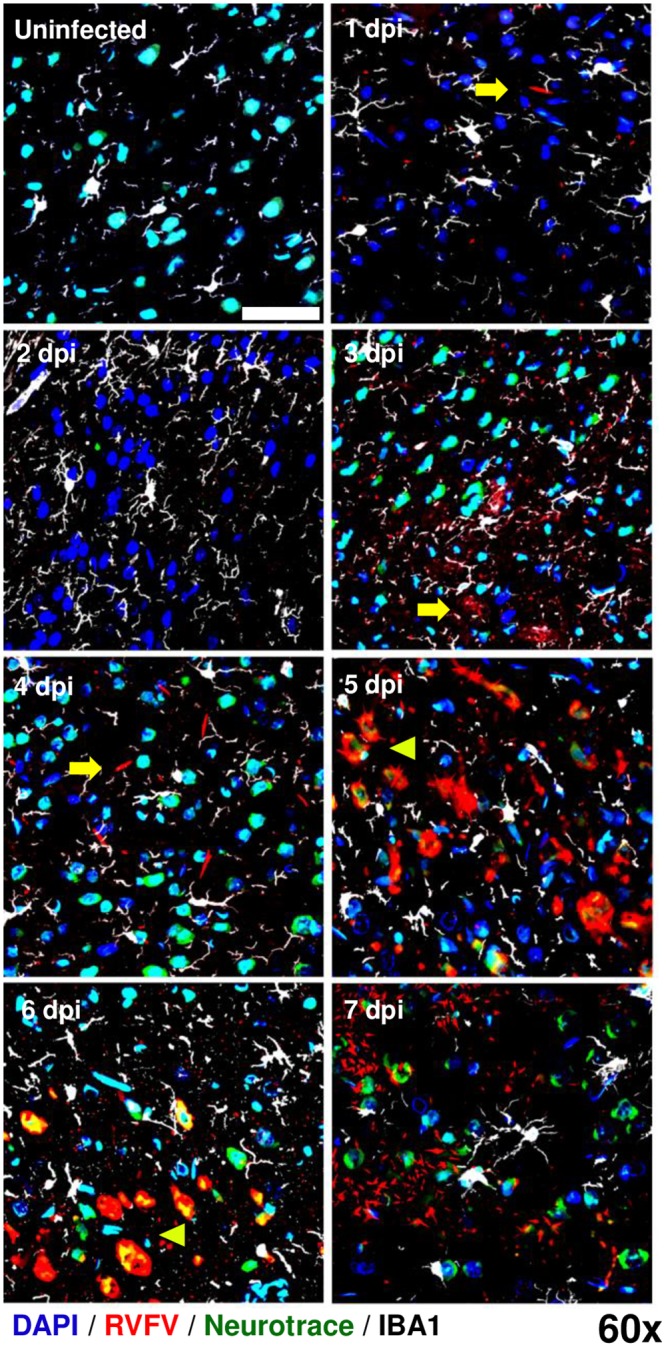
Visualization of RVFV infection in the cortex. AERO-infected Lewis rat brain prefrontal cortexes show evidence of increased RVFV viral RNA over the time course of infection from 1 dpi to 7 dpi. RVFV RNA was detected by ISH-IF (red). Samples were co-stained with an antibody for microglia (IBA-1; white), a Nissl body dye (Neurotrace; green), and nuclear counterstain (DAPI; blue). Yellow arrowheads highlight areas of extracellular virus; yellow pointers highlight infected neurons. Single field 60x magnification images taken with a Nikon A1 confocal microscope (scale bar = 50um).

We used immunofluorescence and confocal microscopy to confirm the immune cell infiltration and infected cells within the olfactory bulb and cerebral cortex of AERO-infected rats. vRNA was detected using an *in situ* hybridization probe (RNAscope) specific for a region within the RVFV N protein. At 1 dpi, vRNA appeared within the glomerular layer of the olfactory bulb and cortex (Figs 7 and 8). Detection of vRNA increased between 3 and 7 dpi originating within the glomerular cell layer and gradually expanding into the mitral and granule cell layers of the olfactory bulb (Fig 7A).

The first evidence of detectable infection of neuronal cell bodies occurred at 3 dpi in the olfactory bulb (Fig 7B, yellow cells represent colocalization of green Neurotrace and red vRNA signal). Within the olfactory bulb, initial infection of neurons was contained to single cells within the glomerular layer, indicating limited viral dissemination detectable by this methodology during early infection (Fig 7A). By 5 dpi, the neurons lining the mitral cell layer co-localized more frequently with vRNA and by 6–7 dpi, the entirety of the remaining neuronal population was positive for vRNA. Neurotrace and vRNA was found within Iba-1+ cells by 6–7 dpi, highlighting extensive neuronal death and phagocytosis of debris by the microglia (Fig 7A and 7B). In the cortex, virus was primarily extra-neuronal until 5 dpi, after which virus-infected neurons were evident. By 7 dpi, the vRNA staining appeared fragmented indicating massive cell death (Fig 8).

Overall, the frequency of Iba-1+ cells was elevated in both olfactory bulb and cortex at all points after infection compared to an uninfected brain (note increased visualization of white microglia in Figs 7 and 8), reflecting possible microgliosis in response to infection. By 4–7 dpi the Iba-1+ cell numbers visually decreased, and remaining Iba-1+ cells, particularly in the olfactory bulb, exhibited the larger, ameboid cell body with few projections compared to the ramified structure of quiescent microglia. These data are congruent with flow cytometry results in Fig 3D.

### Visualization of leukocyte infiltration into the brains of AERO-infected rats

**Fig 9:**
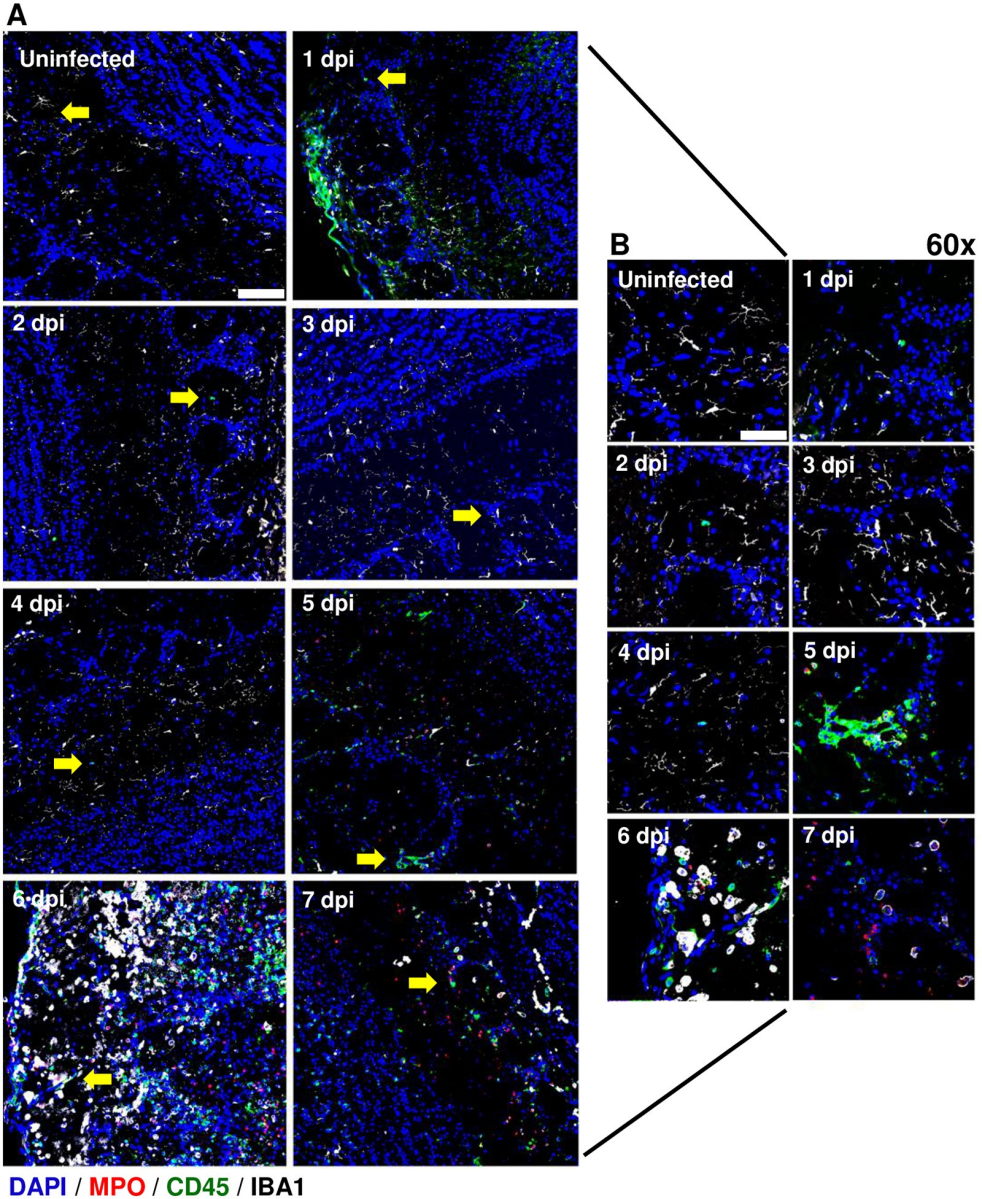
Visualization of leukocyte infiltration into the olfactory bulb of AERO infected rats. AERO-infected Lewis rat olfactory bulbs show evidence of increased cell infiltration from 1 dpi to 7 dpi. Samples were stained with primary antibodies to detect neutrophils (MPO; red), a pan-leukocyte marker (CD45; green), and microglia (IBA-1; white) along with nuclear counterstain (DAPI; blue). (A) 2x2 field images were taken with at 40x magnification and stitched using Nikon Elements software (scale bar = 100um). Yellow arrows indicate areas magnified in (B). (B) Max intensity projection images of the olfactory bulb taken at 60 (scale bar = 50um).

**Fig 10:**
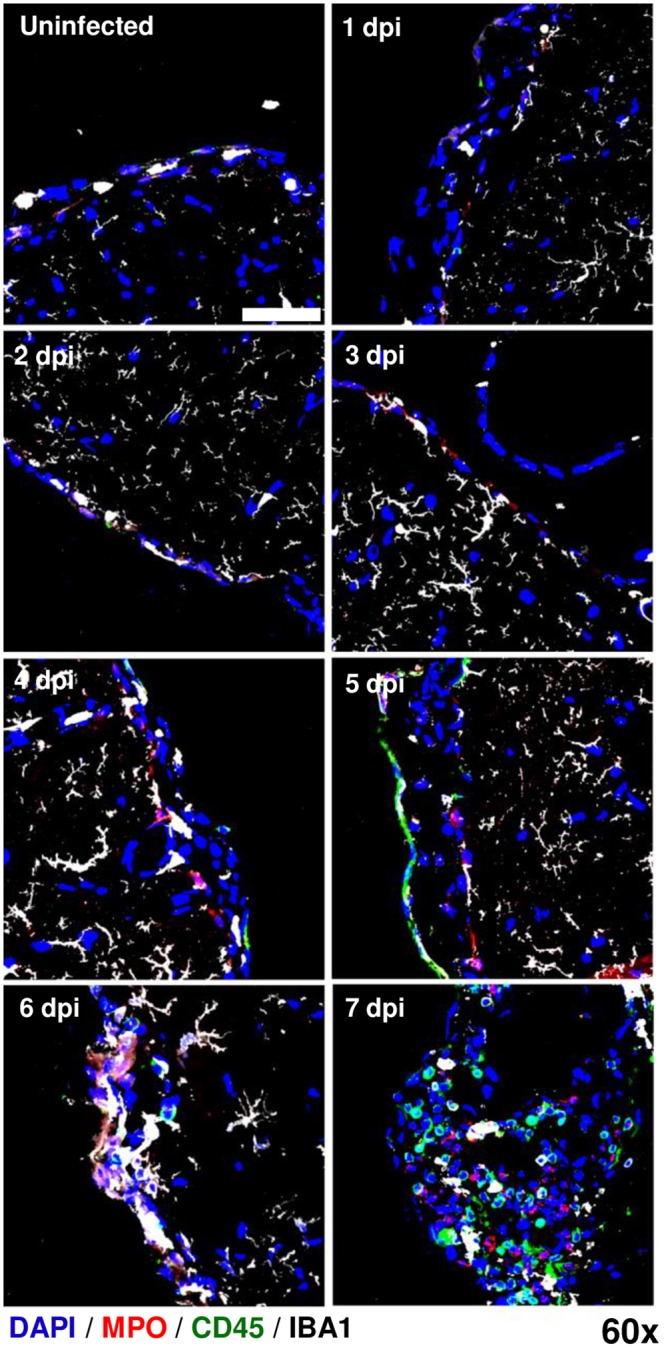
Leukocyte infiltration into the cortex after RVFV AERO infection. RVFV-infected Lewis rat cortexes show evidence of increased cell infiltration from 1 dpi to 7 dpi. Samples were stained with primary antibodies to detect neutrophils (MPO; red), general leukocytes (CD45; green), and microglia (IBA-1; white) along with nuclear counterstain (DAPI; blue). Max intensity projection images of the cortex blood brain barrier taken at 60x (scale bar = 50um).

A second immunofluorescence staining panel was used to visualize leukocyte infiltration within rat brains. Myeloperoxidase (which identifies neutrophils), CD45, and Iba-1 were used to phenotype leukocytes. Data from Fig 3 suggested a possible early influx of leukocytes at 1 dpi that was not associated with massive breakdown of the blood brain barrier [17]. Here, we confirmed early CD45+ cells in the glomerular layer of the olfactory bulb and the surface of the cortex at 1 dpi (Fig 9A and 9B; Fig 10); this is congruent with the flow cytometry results in Fig 3. By 5 dpi, extensive immune cell infiltration was found in both the olfactory bulb and cortex, which fits with our previous study that found breakdown of the brain vasculature at 5 dpi onward [17]. The early CD45+ cell infiltration at 1 dpi also occurs at the same time that we detected the presence of vRNA (Fig 7).

Myeloperoxidase (MPO) is an antimicrobial enzyme expressed primarily by neutrophils and can serve as an effective marker for the presence of neutrophils in tissues. MPO+ cells were found in the cortex, but not olfactory bulb, as early as 1 dpi (Fig 10), with the number of MPO+ cells increasing from 4 dpi onwards. MPO+ cells were first detectable in the olfactory bulb at 5 dpi, corresponding to an increase in overall CD45+ cells (Fig 9). The arrival of MPO+ cells in both the olfactory bulb and cortex by 4–5 dpi coincided with the breakdown of brain vascular integrity [17] and visualization of amoeboid, non-ramified Iba-1+ cells (likely a combination of primarily activated microglia with some infiltrating macrophages; Fig 3). Taken together, our data suggest early leukocyte infiltration together with transient increased permeability may play a role in the pathogenesis of RVFV-induced encephalitis.

### Prevention of neutrophil migration to the brain does not affect survival of rats

Given the extent of neutrophil infiltration and the levels of inflammatory cytokines and chemokines found in the brain of AERO-infected rats, we hypothesize that neutrophils may play a pathogenic role in lethal RVF encephalitis. To prevent neutrophil migration into the CNS, we used a CXC chemokine receptor 2 (CXCR2) antagonist, which has been shown to prevent neutrophil migration to the brain [25]. The chemokine Gro-KC in rats (CXCL1; related to IL-8 in humans) attracts neutrophils, utilizes CXCR2 as its receptor, and was found in high levels in the brains of rats dying from RVF encephalitis (Fig 4B) [19].

**Fig 11:**
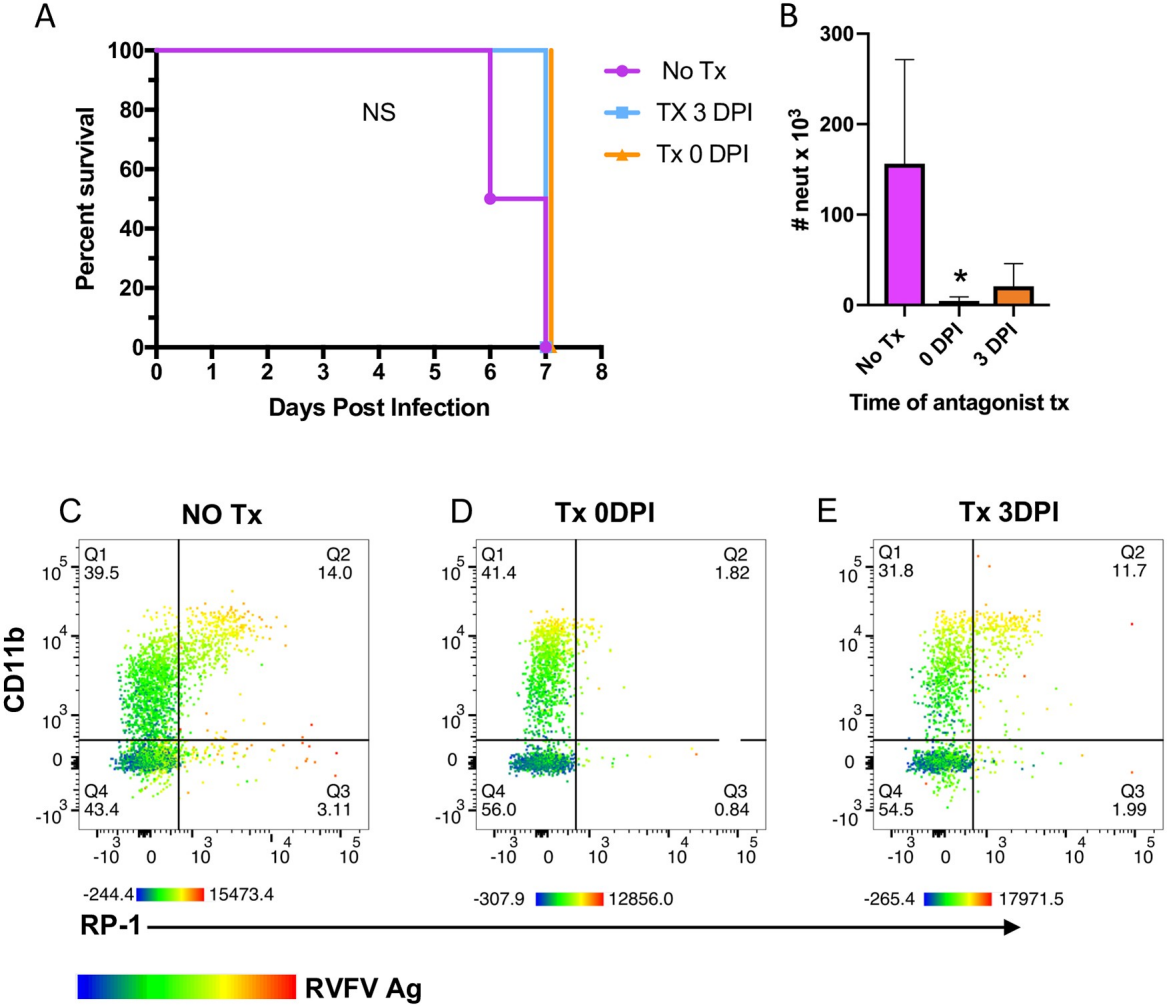
Prevention of neutrophil migration into the brain using a CXCR2 antagonist. RVFV-infected rats were treated with SB-265610 on 0 dpi, 3 dpi, or a vehicle control. (A) Survival curve. Neither 3 dpi nor 0 dpi were significantly different from vehicle control rats using Log-rank Mantel-Cox test. (B) Number of neutrophils in rat brains at time of necropsy as measured by flow cytometry (n = 3 rats/tx group; n = 2 for no tx group). One-way ANOVA with multiple comparisons was used to determine statistical significance. Representative flow plots of neutrophils (RP-1+, CD11b+, CD45+) for (C) no treatment (no Tx) (D) 0 dpi, and (E) 3 dpi treatment groups. Color gradient in B-D indicates RVFV antigen expression, with red indicating the brightest expression and blue the lowest.

Rats were treated with the CXCR2 antagonist at the time of infection (0 dpi) or at 3 dpi prior to neutrophil infiltration into the brain. Survival and time to euthanasia was not different between the infected, untreated controls and either treatment group (Fig 11A). There were also no differences in clinical manifestations such as weight loss, temperature or neurological disease. Flow cytometry on brains from the rats at euthanasia showed a significant reduction in neutrophils in the brain of rats treated at 0 dpi and a partial reduction in those treated at 3 dpi (Fig 11B–11E). Of the remaining cells in the brain, those expressing CD11b and RP-1 were found to be infected, as measured by intracellular antigen staining. Taken together this suggests that preventing neutrophil infiltration into the brain is not enough to alter the outcome of disease in Lewis rats infected with RVFV.

## Discussion

Due to very limited human autopsies samples, little is known about the pathology that occurs within the brain of humans that succumb to RVFV infection. One study found macrophages and lymphocytes in the brain of a patient that died of encephalitis [9]. The susceptibility of human macrophages to RVF infection and their role during in RVF infection in animal models is fairly well-defined in both *in vitro* as well as *in vivo* [26, 27]. Limited studies have focused on the role neutrophil-mediated pathology during RVFV infection compared to macrophages. Polymorphonuclear cells were observed in infected calves, immunocompetent and immunosuppressed mice, young gerbils, and rats in the liver, spleen, and brain [21, 28–32]. Documentation of neutrophils in these previous studies was accomplished primarily by H&E stained tissues. Neutrophils can have a paradoxical role during West Nile Virus infections [33]. For instance, neutrophils are permissive to infection and appear to serve as an early amplifier of virus, whereas late in infection, neutrophils have a protective role in viral clearance [34]. In a mouse model of St. Louis encephalitis, neutrophils were heavily recruited into the brains [35].

Lewis rats are a unique model for understanding RVF neuropathogenesis because of their susceptibility to AERO infection yet they are resistant to disease after SC injection. To our knowledge, this is the first study to directly compare the difference in virus infection and spread after both routes of infection in rats. In a previous study using BALB/c mice, both SC and AERO exposure resulted in lethal hepatic disease in 70–75% of the animals, with the remaining 25–30% developing encephalitis [15]. In the AERO-exposed mice, neurological signs and death occurred 1 day earlier than the SC-infected mice [15].

One may be tempted to hypothesize, as we did, that AERO infection provides a conduit for the virus to travel directly through the nasal neuroepithelium to the olfactory bulb, whereas SC injection of the virus would not result in virus entry into the brain, and hence the rats survive with no disease. The data presented here suggest that the hypothesis regarding SC infection is not correct. We repeatedly detected viral RNA in the brain after SC infection, implying that the difference in outcome between the two routes seems to be dependent on limited viral replication in the brain after SC infection. To support the detection of viral RNA in the brains of SC-infected rats, we also found changes in leukocyte populations within these brains, albeit much less dramatic than after AERO infection. Interestingly, an influx of T cells was detected in the rats that survived, suggesting a possible protective role for these cells during SC infection. A recent study in mice showed that depletion of CD4 or CD8 cells led to increases in the frequency of encephalitis [36]. How virus reaches the brain after SC infection, and how it is subsequently controlled, is not known and is currently under investigation. For alphaviruses, vRNA persists in the brains of infected mice for months after infection, and in some cases the virus is infectious [37, 38]. The persistence of vRNA, and potentially infectious virus, in RVFV-infected rats has implications for a potential unrecognized reservoir in the central nervous system.

In this study, we used complementary approaches of flow cytometry and fluorescence microscopy with *in situ* hybridization to longitudinally identify and quantify the leukocyte infiltration into the olfactory bulb and cerebral cortexes of RVFV-infected rats. Both methods identified an early small influx of neutrophils and other leukocytes at 1 dpi, followed by a more substantial increase from 5 dpi onwards. The CD45^hi^, CD45^med^, and neutrophil populations co-localized with viral antigen; however, we are not able to definitively say whether these cells are productively infected or are co-localizing with viral antigen due to phagocytosis of cell debris.

Distinguishing resident microglia in the brain from infiltrating macrophages is possible using differential expression of CD45, which is expressed at low levels on resting, resident microglia in a normal rat brain [23]. In contrast, high levels of CD45 are expressed on activated microglia and infiltrating peripheral macrophages. At 1 dpi, the AERO-infected rats have transient increases in total numbers of CD45^med^ cells and neutrophils. This was visible with the increase in Iba-1+ cells within the brain microscopy images. This early influx in neutrophils and proliferation of microglia may be detrimental, possibly bringing the virus with it or initiating an inflammatory process. Later in infection, neutrophils, and to a lesser degree macrophages, infiltrate the brain in large numbers, concomitant with the opening of the blood brain barrier and signs of disease in the animals [17].

The late influx of neutrophils and macrophages in the brain corresponded with high levels of the chemokines Gro/KC, MCP-1, MIP-1α, MIP-3α, IL-1β, and IL-1α in brain tissue. Four of these cytokines/chemokines (IL-8/Gro/KC, MCP-1, IL-1α, and MIP-1α) were also elevated in the brains of African green monkeys lethally infected with RVFV by aerosol [20]. This panel of cytokines and chemokines would exacerbate neutrophilic and monocytic inflammation, and could also contribute to microglia activation and recruitment [39, 40].

African green monkeys that survive inhalational RVFV infection have an early cytokine response in the serum that is absent in lethal infections [20]. We found here that sub-lethal SC-infected rats have an early Th2 and Th17-like cytokine response in the serum that is, again, absent during lethal AERO infection. Taken together, a robust cytokine response in the serum within the first few days of infection was associated with sub-lethal infection and survival. Lack of an effective serum cytokine response and inflammatory mediator production in the brain were associated with lethal infection.

Given the influx of neutrophils that we observed, we blocked recruitment of neutrophils to determine the effect on survival. Treatment of rats with CXCR2 antagonist prevented neutrophil influx into the brains. Despite this, the rats still succumbed to disease. We surmise that by the time the brain vasculature is largely permeable at 5 dpi, the neuronal damage from virus destruction is likely too much for effective recovery [17, 19]. In a mouse model of RVF encephalitis, the CNS damage caused by RVFV appears to be primarily virus-mediated rather than immune-mediated [36]. This study demonstrates that RVF encephalitis in rats is not mediated by neutrophils, but we are unable to dismiss the role of microglia and macrophages in immune-mediated CNS damage.

Worldwide emergence and spread of viruses such as West Nile, Chikungunya, and Zika viruses put a spotlight on the threat that emerging mosquito-borne viruses can pose to humans. RVFV is most recognized for its ability to cause hemorrhagic fever in people. However, a substantial number of infected people develop neurological complications as a result of RVFV infection [8]. Encephalitis caused by RVFV is hard to prevent and overcome using traditional vaccines and therapeutics [11, 13–15]. This study expands our understanding of the pathogenic mechanisms of RVF encephalitis and provides a framework for the rational design of therapeutic drugs that will prevent this devastating clinical outcome.

## Materials and methods

### Ethics

This work was approved by the University of Pittsburgh IACUC under protocol #17040334 and #17111713. All animal work was conducted in accordance with the recommendations in the *Guide for the Care and Use of Laboratory Animals* of the National Resource Council. All animals were housed and fed in an Association for Assessment and Accreditation of Laboratory Animal Care (AAALAC)-accredited facility. IACUC-approved euthanasia criteria were based on weight loss, fever, and morbidity.

### Biosafety

All experiments with Rift Valley fever Virus were conducted in the Center for Vaccine Research (CVR) and the Regional Biosafety Laboratory (RBL) at the University of Pittsburgh following the safety procedures described previously [16]. The RBL is a registered BSL-3/ABSL-3 laboratory space with the CDC and USDA.

### Virus and cells

The ZH501 strain of Rift Valley fever virus used in these experiments was generously provided by Barry Miller (CDC, Fort Collins, Colorado) and Stuart Nichol (CDC, Atlanta, Georgia) as described previously [16]. Vero E6 (CRL-1586, American Type Culture Collection) cells were used to propagate virus following standard cell culture conditions in Dulbecco’s modified Eagle’s medium (DMEM) containing 2% or 10% fetal bovine serum (FBS), 1% penicillin-streptomycin (pen/strep), and 1% L-glutamine. HAPI (highly aggressive proliferating immortalized) rat microglia (ATCC CRL-2815) were also cultured in standard DMEM. HMC-3 human microglia (HMC-3; ATCC CRL-3304) were cultured using DMEM supplemented with 12% FBS and 1% pen/strep. SH-SY5Y neuroblastoma cells (ATCC CRL-2266) were cultured using 1:1 mixture of DMEM and F12 media, supplemented with 10% FBS, 1% pen/strep, and 1% L-glutamine. SH-SY5Y cells were differentiated using 10uM retinoic acid in neurobasal media supplemented with B27, with media change every 48 hours. For quantitation, virus was measured using previously described methods of viral plaque assay and taqman q-RT-PCR [19].

For virus isolation experiments, 100 ul of clarified cortex homogenate was added to Vero E6 cells in 6-well plates. After the adsorption period, the inoculum was not removed and 2 ml of virus growth media (DMEM described above with 2% FBS) was added on top. Plates were observed daily for CPE. At 5 dpi, all of the supernatant from each well was transferred to a T25 flask of confluent Vero E6 cells, and CPE was monitored for 7 days. After 7 days, the supernatant was harvested for measurement of vRNA by q-RT-PCR as described.

### Rat experiments

All animal work conducted was reviewed and approved by the University of Pittsburgh IACUC. Female Lewis rats (LEW/SsNHsd) rats were obtained from Harlan Laboratories between 8–10 weeks of age. The data presented in this manuscript represent a compilation of samples from several independent serial sacrifice experiments. The doses used in these studies (3x10^4^ pfu for AERO and 1x10^5^ pfu for SC infection) are the actual presented doses for AERO and SC groups, not the intended doses. The presented doses were determined by performing plaque assays on the material injected into the SC rats, and also sampling the air during the aerosol exposure and then performing plaque assay to calculate the presented dose [41]. The intended dose for each group was 1x10^5^ pfu/rat, and we chose this dose because it does typically not result in death after SC infection yet it is high enough to be able to compare to AERO infection; this is also an SC challenge dose commonly used for RVFV challenge studies in mice [31, 42]. We also chose to use high intended dose because we wanted to give the aerosol-infected rats a high enough dose to ensure simultaneous development of disease within the 7 day time frame in order for the serial euthanasia experiments to be more consistent since each time point represents a different group of animals. We underachieved the presented dose in the aerosol experiments, and it is not uncommon to be off on the presented dose during aerosol exposure due to inherent variability within the system [41]. For each experiment, 3–4 rats were euthanized per day from 1–6 or 7 dpi to collect tissues. Rats typically reach euthanasia criteria by 7 dpi after AERO exposure to this dose of RVFV.

RVFV ZH501 aerosol infections were performed in a class III aerobiology cabinet as described previously [16]. Subcutaneous injections were performed in the right hind leg (500 ul total volume). The intended target dose for both infection routes was 1x10^5^ pfu/rat. The presented dose, determined by plaque assays from aerosol sampling devices used during the aerosol exposure [41], was 3x10^4^ pfu/rat. The presented dose for SC exposed animals, as determine by plaque assay on the material injected into the animals, was 1x10^5^ pfu/rat. After infection, temperature and weights were taken daily in addition to observation for any clinical signs of illness. Rats were euthanized daily from 1–6 or 7 dpi. Immediately before euthanasia, blood was drawn by cardiac puncture and saved for analysis including complete blood count blood chemistry using the Abaxis HM2 and VS2, respectively. For CBC analysis, data from rats infected SC with 1x10^5^ pfu/rat and euthanized at 10 dpi is included (Fig 2). For experiments presented in Figs 7–10, rats were perfused with PBS followed by 4% paraformaldehyde (PFA) prior to organ collection for imaging analysis.

### Chemicals

SB-265610 (Sigma Aldrich SML0421) was suspended in DMSO and diluted in sterile Dulbecco’s PBS on the day of injection. SB-265610 (2 mg/kg) was administered interperitoneally once daily in a volume of 300 ul.

### Whole blood processing

Whole blood (50 ul) was taken from each animal and directly stained with the antibodies diluted in FACS buffer for 15 minutes. Antibodies used were all from BD Biosciences unless otherwise indicated: CD45 (OX-1), HIS-48 (His-48), RP-1 (RP-1) CD11b (WT.5), CD3 (1FA), CD4 (OX-35), CD8 (OX-8), CD163 (His-36), CD68 (ED1), Iba-1 (Abcam; EPR6136), and RVFV monoclonal (BEI; NR-43195). 450uL of FACS lysis buffer was used to lyse red blood cells for 30 minutes. Samples were then washed twice with FACS buffer and fixed in 4% PFA. Gating strategy for whole blood samples is shown in S1 Fig.

### Brain cell isolation and flow cytometry

After perfusion of rats with saline, whole brains were removed immediately for cell isolation essentially as described [23]. Brains were divided into hemispheres, covered with digestion buffer consisting of modified HBSS without calcium and magnesium, 10mg/ml DNase I (Sigma 10104159001), 20mg/ml of collagenase (Sigma C2674) and mechanically digested using a scalpel. The sample was then incubated at 37°C for 45 minutes on a continuous rocker. Every 15 minutes, the sample was mechanically triturated using a serological pipet. The resulting homogenate was then filtered through a 40 um cell strainer and washed twice with wash buffer, consisting of HBSS with 3% FBS and 10 mg/ml DNase I and centrifuged at 500 x g for 8 minutes at room temperature. The supernatant was removed and the remaining pellet was suspended in 80% stock isotonic Percoll (SIP) (Sigma GE17-0891-01) made in HBSS solution. The suspension was subsequently overlaid with 10 ml of 38% SIP, 10 ml 21% SIP, followed by 5 ml HBSS with 3% FBS and centrifuged at 480 x gravity for 35 minutes, no brake. The third interface was removed, washed twice with modified HBSS containing 3% FBS and then suspended in 1 ml FACS buffer. Cells were counted using a hemocytometer and placed on ice. Cells suspended in FACS buffer were placed in a V-bottom 96-well plate and centrifuged for 500 x gravity for 4 minutes at 4°C. An Fc block was performed by adding 2uL of purified anti-CD32 (BD Biosciences) and 18uL of FACS buffer per sample for 20 minutes on ice in the dark. Cells were then washed twice with 200uL FACS buffer and stained with live/dead. Cells were washed with 200uL of FACS buffer and then stained antibody mix for 30 minutes on ice in the dark. Antibodies and clones are listed above. Samples that required intracellular stain were permeabilized and fixed using BD Cytofix/Cytoperm (BD554714) and then washed with FACS perm-buffer. The stained samples were then washed twice with 200uL FACS buffer followed by fixation with 200uL of 4% PFA. Samples were run on a BD LSRII and analyzed using FlowJo 10.5.0. Cell analysis began with a vital brain gate using FSC and SSC, followed by live/dead, then singlet inclusion using SSC-A and SSC-H. From there, CD45 expression levels were determined using SSC-A, and the gating strategy in S2 Fig was followed.

### Cytokine measurements

50uL of clarified rat brain cortex homogenate or rat serum was run on Bio-Plex Pro Rat Cytokine 23-plex assay (BioRad #12005641) following the kits instructions. At the time of purchase of the kits, only 22 parameters were available. The 22 parameters measured were: G-CSF, GM-CSF, GRO/KC, IFN-γ, IL-1α, IL-1β, IL-2, IL-4, IL-5, IL-6, IL-7, IL-10, IL-12 (p70), IL-13, IL-17A, IL-18, M-CSF, MCP-1, MIP-1α, MIP-3α, RANTES, TNF-α, VEGF. Samples were analyzed on Bio-Plex 200 system located within BSL-3 containment. Cytokines not shown in Fig 4 are shown in S1 and S2 Figs.

### In situ hybridization (ISH) and immunofluorescence (IF)

For fixation of tissues and inactivation of virus, rats were perfused with PBS followed by 4% PFA. Brain tissue was extracted and submerged in 4% PFA for 3 hours at 4°C, followed by 30% sucrose and kept at 4°C for 1 week before flash freezing with 2-methylbutane and liquid nitrogen. Frozen samples were cryo-sectioned at 7um thickness. Antibodies used for IF include: goat anti-IBA1 (Novus; NB100-1028), Rabbit anti-MPO (Abcam; ab9535), mouse anti-CD45-647 (BD Pharmigen; 565465), neurotrace-640 (Invitrogen; N21482). Secondary antibodies include: donkey anti-goat Alexa Fluor 488 (Invitrogen), Cy5 Affinipure donkey anti-mouse IgG (Jackson ImmunoResearch), Cy3 Affinipure donkey anti-rabbit IgG (Jackson ImmunoResearch).

For visualization of viral RNA and cell surface markers: Slides were permeablized for 30 minutes using 0.1% TritonX100 + 1x PBS at RT. The protease step in RNAscope kit was omitted to preserve microglia and neuronal antigen. Slides were stained in slide box lined with wet paper towels and placed in an incubator at 37°C rather than RNAscope hybridization oven. RNAscope kit was, otherwise, used according to manufacturer’s instructions with RVFV ZH501 probe against NP gene (Cat No. 496931). Primary antibody for IBA1 or Neurotrace-640 were incubated for 1 hour at RT. Secondary antibody (was incubated for 1 hour at RT. Hoescht (Bis-benzamide) was incubated for 1 minute to counterstain nuclei. Samples were mounted using a glycerol + PVA mixture.

For visualization of immune cell infiltration using IF: Slides were permeabilized for 30 minutes using 0.1% TritonX100 + 1x PBS at RT. Slides were incubated with 5% normal donkey serum diluted with 0.5% BSA + 1x PBS to block nonspecific binding of the secondary antibodies for 45 minutes. Primary antibodies were incubated for 1 hour at 4°C. Secondary antibodies were incubated for 1 hour at RT. Hoescht (Bis-benzamide) was incubated for 1 minute to counterstain nuclei. Samples were mounted using a glycerol + PVA mixture.

Slides were imaged using the Nikon A1 Confocal Microscope provided by the Center for Biologic Imaging. Images were contrasted using Adobe Photoshop and de-noised and analyzed using Nikon Elements.

### Statistics

Statistical analyses were performed using GraphPad Prism software (La Jolla, CA). For Figs 2,3 and 4, S1, and S2, two-way analysis of variance (ANOVA) was performed, with the 2 factors being exposure route (AERO vs SC) and time (0–7 dpi). Dunnett’s multiple comparison test was also performed, which compared the mean value for each day post-infection of each exposure route to the mean of the pre-infection control sample. P values for each comparison were adjusted to account for multiple comparisons. On each graph, the # in the bottom right corner of the graph indicates the p-value for the comparison of SC vs AERO exposure route (N.S., not significant; #, P < 0.05; ##, P < 0.01; ###, P < 0.001; ####, P < 0.0001). Asterisks above symbols indicate significant differences at a particular time point within each exposure route compared to uninfected samples (*, P < 0.05; **, P < 0.01; ***, P < 0.001; ****, P < 0.0001) as determined by Dunnett’s multiple-comparison tests. Asterisks are color coded by exposure route group. For Figs 5A and 11B, one-way ANOVA was used to determine statistical significance between the groups at each time point (*, P < 0.05; **, P < 0.01; ***, P < 0.001; ****, P < 0.0001). For Fig 11A, Log-rank Mantel-Cox test was used to determine differences in survival with and without CXCR2 antagonist treatment.

## Supporting information

S1 Fig Serum cytokine responses. Cytokines were assessed in serum by a 22-plex rat-specific Luminex kit. CTL represents mock-infected rats. For statistical analysis, 2-way ANOVA with multiple comparisons was performed (see Methods section). The # in the bottom right corner indicates significance by 2-way ANOVA (N.S., not significant; #, P < 0.05; ##, P < 0.01; ###, P < 0.001; ####, P < 0.0001); asterisks above symbols indicate significance of individual time points compared to uninfected (*, P < 0.05; **, P < 0.01; ***, P < 0.001; ****, P < 0.0001). Asterisk above a bar indicates significance over the encompassed data points.
(PPTX)

S2 Fig Brain cytokine responses. Cytokines were assessed in homogenized brain tissue by a 22-plex rat-specific Luminex kit. CTL represents mock-infected rats. For statistical analysis, 2-way ANOVA with multiple comparisons was performed (see Methods section). The # in the bottom right corner indicates significance by 2-way ANOVA (N.S., not significant; #, P < 0.05; ##, P < 0.01; ###, P < 0.001; ####, P < 0.0001); asterisks above symbols indicate significance of individual time points compared to uninfected (*, P < 0.05; **, P < 0.01; ***, P < 0.001; ****, P < 0.0001). Asterisk above a bar indicates significance over the encompassed data points.
(PPTX)

S3 Fig Gating strategy for whole blood samples. Representative whole blood sample from AERO-infected rat at 7 dpi. (A) SSC-A and FSC-A. Gating was first done using (B) singlet inclusion, followed by (C) CD45+ gate and (D) size exclusion.
(PPTX)

S4 Fig Gating strategy for brain samples. Representative brain sample from AERO-infected rat at 7 dpi. General gating strategy begins with (A) vital brain gate, (B) live/dead gating and (C) singlet inclusion. Myeloid cells were gated as follows: (D) Peripheral macrophages as CD163+, (E) neutrophils as RP-1+ CD11b+, and (F) microglia as Iba-1+. (G) Iba1+ microglia can be further characterized based on level of CD45 expression. T-cells were gated as (H) CD3+ and then categorized as either (I) CD8+ or CD4+.
(PPTX)
