# Supplementary material for: Neutrophil and macrophage influx into the central nervous system are inflammatory components of lethal Rift Valley fever encephalitis in rats
Source: PLoS Pathog. 2019 Jun 20;15(6):e1007833. doi: 10.1371/journal.ppat.1007833 (PMC6605717; doi:10.1371/journal.ppat.1007833)
Supplement: S1 Fig — Cytokines were assessed in serum by a 22-plex rat-specific Luminex kit. CTL represents mock-infected rats. For statistical analysis, 2-way ANOVA with multiple comparisons was performed (see Methods section). The # in the bottom right corner indicates significance by 2-way ANOVA (N.S., not significant; #, P < 0.05; ##, P < 0.01; ###, P < 0.001; ####, P < 0.0001); asterisks above symbols indicate significance of individual time points compared to uninfected (*, P < 0.05; **, P < 0.01; ***, P < 0.001; ****, P < 0.0001). Asterisk above a bar indicates significance over the encompassed data points. (PPTX) [file ppat.1007833.s001.pptx]

## Slide 1
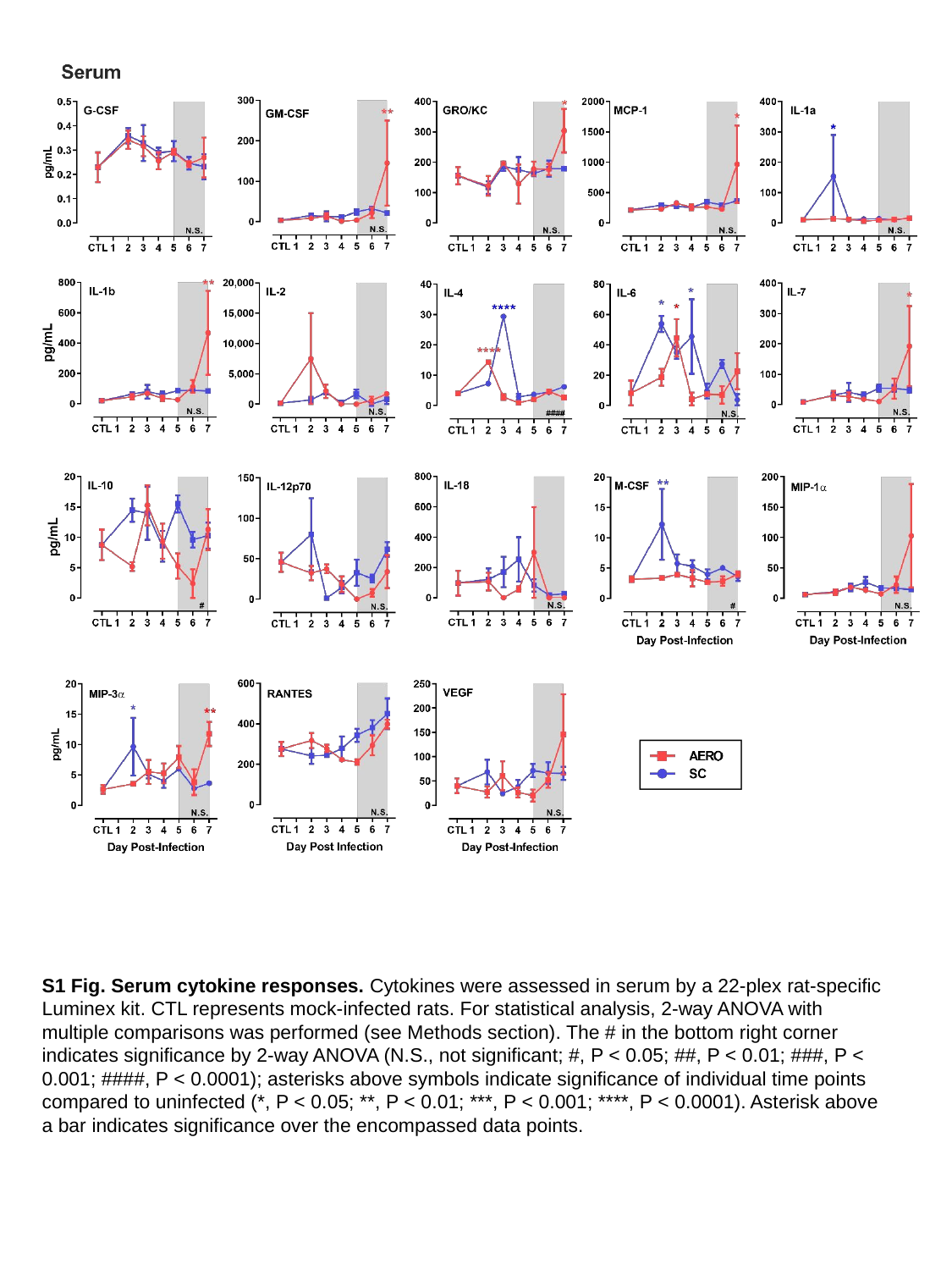

S1 Fig. Serum cytokine responses. Cytokines were assessed in serum by a 22-plex rat-specific Luminex kit. CTL represents mock-infected rats. For statistical analysis, 2-way ANOVA with multiple comparisons was performed (see Methods section). The # in the bottom right corner indicates significance by 2-way ANOVA (N.S., not significant; #, P < 0.05; ##, P < 0.01; ###, P < 0.001; ####, P < 0.0001); asterisks above symbols indicate significance of individual time points compared to uninfected (*, P < 0.05; **, P < 0.01; ***, P < 0.001; ****, P < 0.0001). Asterisk above a bar indicates significance over the encompassed data points.
