# Supplementary material for: Neutrophil and macrophage influx into the central nervous system are inflammatory components of lethal Rift Valley fever encephalitis in rats
Source: PLoS Pathog. 2019 Jun 20;15(6):e1007833. doi: 10.1371/journal.ppat.1007833 (PMC6605717; doi:10.1371/journal.ppat.1007833)
Supplement: S3 Fig — Representative whole blood sample from AERO-infected rat at 7 dpi. (A) SSC-A and FSC-A. Gating was first done using (B) singlet inclusion, followed by (C) CD45+ gate and (D) size exclusion. (PPTX) [file ppat.1007833.s003.pptx]

## Slide 1
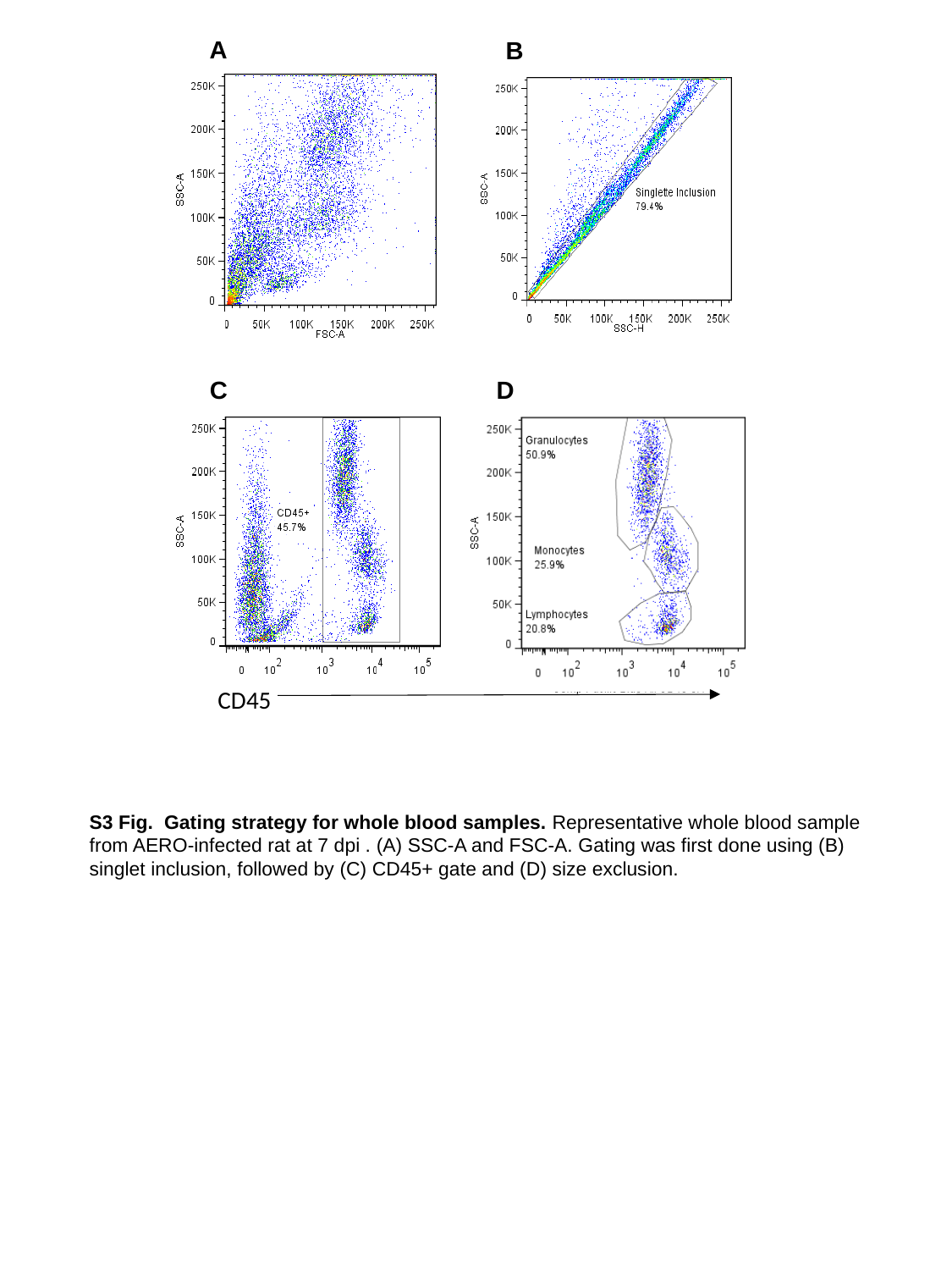

A
B
C
D
CD45
S3 Fig. Gating strategy for whole blood samples. Representative whole blood sample from AERO-infected rat at 7 dpi . (A) SSC-A and FSC-A. Gating was first done using (B) singlet inclusion, followed by (C) CD45+ gate and (D) size exclusion.
