# Supplementary material for: Neutrophil and macrophage influx into the central nervous system are inflammatory components of lethal Rift Valley fever encephalitis in rats
Source: PLoS Pathog. 2019 Jun 20;15(6):e1007833. doi: 10.1371/journal.ppat.1007833 (PMC6605717; doi:10.1371/journal.ppat.1007833)
Supplement: S4 Fig — Representative brain sample from AERO-infected rat at 7 dpi. General gating strategy begins with (A) vital brain gate, (B) live/dead gating and (C) singlet inclusion. Myeloid cells were gated as follows: (D) Peripheral macrophages as CD163+, (E) neutrophils as RP-1+ CD11b+, and (F) microglia as Iba-1+. (G) Iba1+ microglia can be further characterized based on level of CD45 expression. T-cells were gated as (H) CD3+ and then categorized as either (I) CD8+ or CD4+. (PPTX) [file ppat.1007833.s004.pptx]

## Slide 1
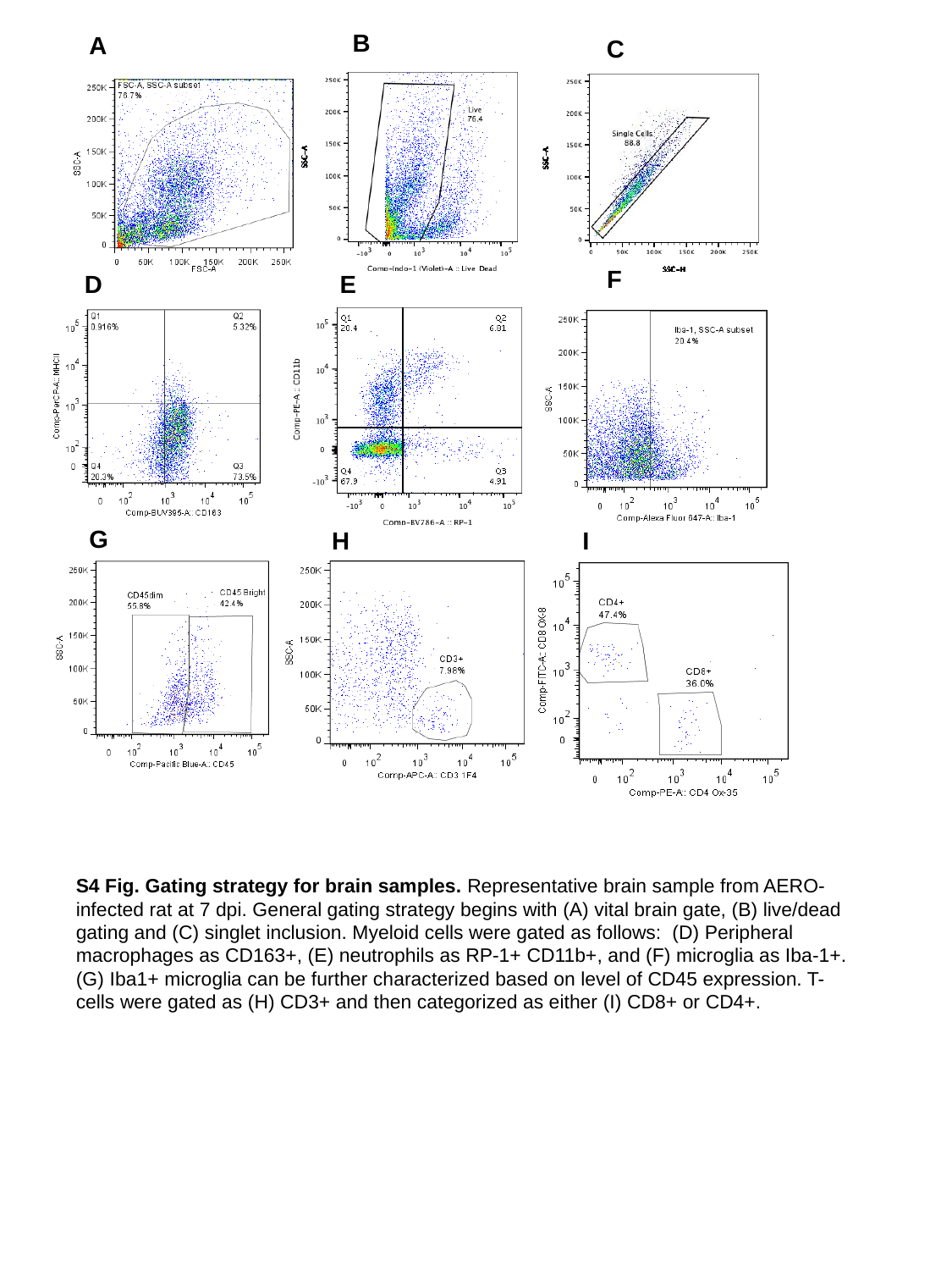

B
A
C
F
E
D
G
H
I
S4 Fig. Gating strategy for brain samples. Representative brain sample from AERO-infected rat at 7 dpi. General gating strategy begins with (A) vital brain gate, (B) live/dead gating and (C) singlet inclusion. Myeloid cells were gated as follows: (D) Peripheral macrophages as CD163+, (E) neutrophils as RP-1+ CD11b+, and (F) microglia as Iba-1+. (G) Iba1+ microglia can be further characterized based on level of CD45 expression. T-cells were gated as (H) CD3+ and then categorized as either (I) CD8+ or CD4+.
